# On Using Simulation to Predict the Performance of Robot Swarms

**DOI:** 10.1038/s41597-022-01895-1

**Published:** 2022-12-29

**Authors:** Antoine Ligot, Mauro Birattari

**Affiliations:** IRIDIA, Université libre de Bruxelles, Brussels, Belgium

**Keywords:** Mechanical engineering, Electrical and electronic engineering

## Abstract

The discrepancy between simulation and reality–known as the reality gap–is one of the main challenges associated with using simulations to design control software for robot swarms. Currently, the reality-gap problem necessitates expensive and time consuming tests on physical robots to reliably assess control software. Predicting real-world performance accurately without recurring to physical experiments would be particularly valuable. In this paper, we compare various simulation-based predictors of the performance of robot swarms that have been proposed in the literature but never evaluated empirically. We consider (1) the classical approach adopted to estimate real-world performance, which relies on the evaluation of control software on the simulation model used in the design process, and (2) some so-called pseudo-reality predictors, which rely on simulation models other than the one used in the design process. To evaluate these predictors, we reuse 1021 instances of control software and their real-world performance gathered from seven previous studies. Results show that the pseudo-reality predictors considered yield more accurate estimates of the real-world performance than the classical approach.

## Introduction

In swarm robotics, the design of the control software that determines a robot’s behavior is typically performed off-line, in simulation, prior to the deployment in the target environment^1–8^. Control software can also be designed on-line—that is, while robots are already operating in the environment—but this approach comports a number of drawbacks with respect to the off-line one^9^. Computer-based simulation is indeed an appealing tool to develop control software for robot swarms—and also for single-robot and non-swarm multi-robot systems—as it allows for fast, safe, and inexpensive evaluations of control software. Furthermore, simulation provides a god’s-eye view that allows evaluating all possible measures of performance, including those that the robots could not evaluate themselves. As a result, simulation enables using off-line automatic design methods, which require multiple evaluations of many instances of control software to converge to one that satisfactorily performs the task at hand. However, designing control software on the basis of a simulation model—which we refer to as the *design model*—has a major drawback: because of the possibly small but unavoidable inaccuracies of the design model with respect to reality, physical robots are likely to display different behaviors from those observed in simulation^10,11^. The discrepancies between simulation and the real world are commonly referred to as the *reality gap*, which is widely understood to be a critical problem in robotics^12–14^.

Due to the reality gap, the performance of control software observed in reality is likely to be lower than the one obtained via evaluations on the design model. By performance, we mean here a measure of the extent to which the swarm is successful in attaining the goals of the given mission. This measure is part of the formal specification of the mission and is what the automatic design process aims to maximize. To give an example, in a foraging mission, the performance could be the number of items retrieved per unit of time. The occurrence of drops in performance is a *relative* problem: some instances of control software are more seriously affected by the reality gap than others. The relative nature of the effects of the reality gap can lead to a situation in which an instance of control software *CS*_*A*_ outperforms another instance *CS*_*B*_ in simulation, but *CS*_*B*_ outperforms *CS*_*A*_ when they are executed on physical robots^15,16^. This phenomenon, which we call a *rank inversion*^17,18^, is particularly insidious as it questions the validity of the off-line design process in that it relies on the assumption that the higher the performance in simulation, the higher the performance in reality.

Several studies have been devoted to proposing approaches for handling the reality gap^18^. These approaches—whether by improving the accuracy of the design model^13,19–22^, or by increasing the robustness of the control software to the differences between the design model and reality^15,23–26^—all aim to produce control software that crosses the reality gap satisfactorily; that is, control software that suffers from the smallest performance drop possible. Unfortunately, the proposed approaches have not been empirically assessed or compared, and none of them appears to be the ultimate solution^14,27^. The reality gap remains an important problem to be faced in robotics, which currently makes costly and time consuming tests on physical robots the only reliable way to assess control software. A simulation procedure to accurately predict real-world performance of control software would be extremely valuable.

**Fig. 1:**
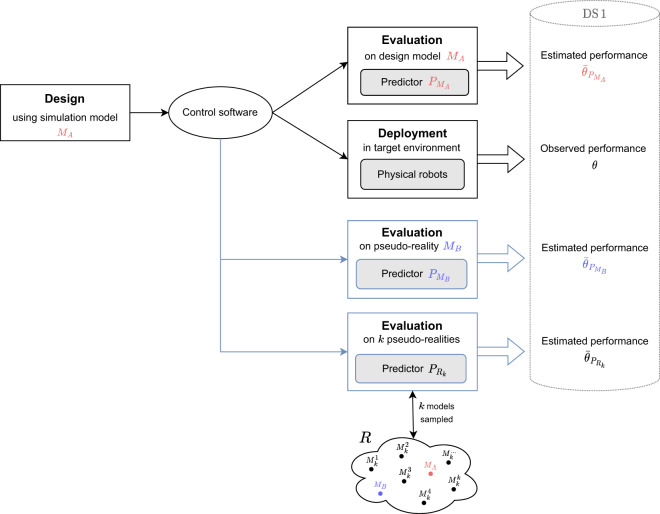
Schematic overview of the study. In black, the typical process for generating control software for robot swarms consisting of a design phase, an evaluation phase, and eventually a deployment phase. The design phase is performed on the basis of a simulation model here named *M*_*A*_. The evaluation phase is performed in simulation, on the same simulation model used during the design, and is a common way of estimating the real-world performance of control software. We name this popular predictor ${P}_{{M}_{A}}$. During the deployment phase, in which control software is executed on physical robots in the target environment, the actual performance *θ* of the control software is observed. In blue, our evaluation of the control software with pseudo-reality predictors. The concept of pseudo-reality, which refers to a simulation model that differs from the one used in the design, emerged from the contention that if an instance of control software shows similar performance in the simulation model on which it has been designed and in a different simulation model (or several ones), it is somehow ‘intrinsically’ robust and it can be expected to cross the reality gap more satisfactorily than another instance that does not. The predictor ${P}_{{M}_{B}}$ evaluates each instance of control software on a single pseudo-reality model named *M*_*B*_. The previously defined predictor ${P}_{{R}_{1}}$ evaluates each of them once on a randomly sampled model from the set *R*, which contains both *M*_*A*_ and *M*_*B*_. We introduce ${P}_{{R}_{k}}$, a generalized version of ${P}_{{R}_{1}}$, which evaluates each instance once on *k* randomly sampled models from *R*, and considered *k* = {1, 3, 5, 10, 30, 50, 100, 500}. The observed performance that we collected from previous studies, as well as the predicted performance given by the predictors we considered, are part of the dataset DS 1. See Methods for details about DS 1 and the predictors.

Roboticists commonly report the performance obtained on the design model, alongside the one observed on physical robots, to show whether the control software crosses the reality gap satisfactorily or not. Evaluations of control software on the design model is typically considered as a natural way to estimate its real-world performance (Fig. 1). However, several studies report cases of severe performance drop and rank inversion, which suggests that the performance observed on the design model does not yield an accurate and reliable prediction of the actual performance that one will eventually obtain in the real world^15,16,22,27–30^. In previous publications, we introduced the notion of *pseudo-reality* to demonstrate that the performance drop due to the reality gap is not necessarily a result of the fact that the design model is a simplified version of the target environment but is rather to be understood as an overfitting problem akin to the one encountered in machine learning^17,18^. A pseudo-reality is a simulation model, different from the one used in the design, whose purpose is indeed to evaluate control software. The concept of pseudo-reality emerged from the contention that an instance of control software that can cross satisfactorily the gap between the design model and a pseudo-reality (or, even better, multiple pseudo-realities) is somehow “intrinsically” robust and is to be expected to be more likely to cross the reality gap than another instance that cannot^18^.

Previously, we have shown that it is possible to create a virtual, simulation-only reality gap between the design model and a pseudo-reality model—which we named *M*_*B*_—that yields a performance drop that is qualitatively similar to one observed in reality^17^–see Methods for details on the procedure followed to define *M*_*B*_. We also obtained results that were qualitatively similar to the ones observed on physical robots by using multiple virtual reality gaps between the design model and pseudo-reality models randomly sampled from a range *R*^18^. In these previous studies, we handpicked the single pseudo-reality model *M*_*B*_ and fine-tuned the range *R* with the goal of mimicking the performance drop of control software produced by two design methods to solve two missions. Although promising, the evidence produced so far is insufficient to elaborate any claim about the accuracy of these pseudo-reality predictors, nor about their superiority with respect to the classical evaluations on the design model. Indeed, in addition to the fact that we only reported qualitative results, these results were obtained on the same data used for the definition of the pseudo-reality predictors, which fails to communicate on their generalization capability. Here, we address these shortcomings: we propose quantitative metrics, and we perform a thorough investigation of whether the reliability of these pseudo-reality predictors generalizes to control software produced by a wider range of methods and on a wider range of missions.

We created DS 1, a dataset of performance of robot swarms assessed on physical robots^31^. We gathered publicly available data—that is, instances of control software and associated real-world performance—from several studies in automatic design of robots swarms^27,32–37^. In total, we collected 1021 instances of control software generated by 18 different off-line design methods for 45 missions^38^. By reusing data collected previously for other purposes—which, to the best of our knowledge, is a premiere in the automatic design of control software for robot swarms—we were able to perform an analysis that would have been quite costly should we had to generate the required data from scratch. All these instances were designed automatically on the basis of the same ARGoS^39^ simulator model—we named this design model *M*_*A*_—and evaluated on swarms of e-puck robots^40^. In addition to the real-world performance of the collected control software, DS 1 also contains the predicted performance obtained by evaluating the collected control software in simulation on the design model *M*_*A*_, the previously defined pseudo-reality model *M*_*B*_^17^, and 1380 pseudo-reality models uniformly sampled from the previously defined range *R*^18^. Evaluations on the models *M*_*A*_ and *M*_*B*_ correspond to the process of obtaining performance forecasts by the predictors we refer to as ${P}_{{M}_{A}}$ and ${P}_{{M}_{B}}$, respectively. Evaluations on the randomly sampled models allows us to execute the process of the previously defined predictor ${P}_{{R}_{1}}$ which consists, for each instance of control software, in sampling a model from the range of possible models *R* and evaluating the instance of control software on it^18^. It also allows us define the predictor ${P}_{{R}_{k}}$, a generalized version of ${P}_{{R}_{1}}$ that consists, for each instance of control software, in sampling *k* models from *R* and evaluating on each of them the instance of control software once. Details about DS 1, the predictors, the automatic design methods and the missions for which the considered control software has been generated are given in Methods. We compare the predicted performance with the one observed on the physical robots, and we assess the accuracy of the predictors ${P}_{{M}_{A}}$, ${P}_{{M}_{B}}$, and ${P}_{{R}_{1}}$ according to three evaluation criteria. We also do so for ${P}_{{R}_{k}}$ with *k* ∈ {1,3,5,10,30,50,100,500}. Results show that the pseudo-reality predictors ${P}_{{M}_{B}}$ and ${P}_{{R}_{k}}$ yield significantly more accurate predictions than the traditional estimations obtained via evaluations on the design model.

## Results

### Prediction of estimated performance: the error

The quantity *error* measures the accuracy of the predictions of the expected real-world performance of control software. We compute the normalized differences between the predicted performance $\bar{\theta }$ and the performance *θ* observed in reality, as reported in DS 1, as

1 $$error={\left(\frac{\bar{\theta }-\theta }{\theta }\right)}^{2}.$$

**Fig. 2:**
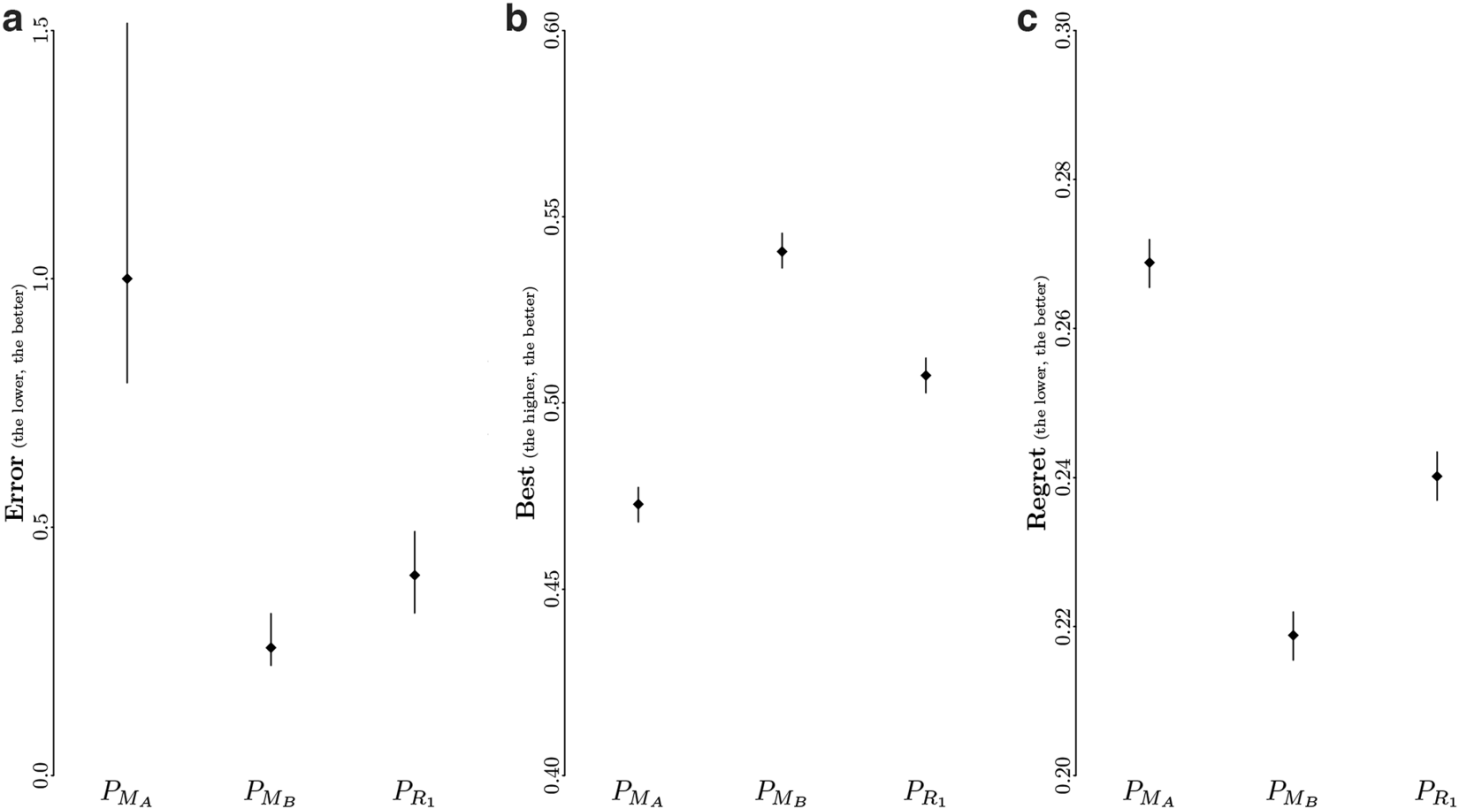
*Error*, *best*, and *regret* of the predictors ${P}_{{M}_{A}}$, ${P}_{{M}_{B}}$, and ${P}_{{R}_{1}}$. In each plot, points represent the (**a**) median *error*, (**b**) mean *best*, and (**c**) mean *regret*; vertical segments represent the respective 95% confidence interval.

Figure 2a reports the median *error* of the predictors ${P}_{{M}_{A}}$, ${P}_{{M}_{B}}$, and ${P}_{{R}_{1}}$. Results show that estimating the expected performance of control software on the basis of evaluations on the same simulation model used in the design process yields less accurate predictions than any of the two other pseudo-reality predictors. In fact, the median *error* of the predictor ${P}_{{M}_{A}}$ is at least 2.4 times greater than the one of the other predictors: ${P}_{{M}_{A}}$ obtains a median error of 1.0, ${P}_{{M}_{B}}$ one of 0.26, and ${P}_{{R}_{1}}$ one of 0.41. Although the two lines representing the 95% confidence interval of the median *error* of ${P}_{{M}_{B}}$ and ${P}_{{R}_{1}}$ are close, they do not overlap. ${P}_{{M}_{B}}$ is therefore significantly more accurate than both ${P}_{{M}_{A}}$ and ${P}_{{R}_{1}}$.

### Prediction of best instance of control software: the best

The quantity *best* measures the ability of predictors to accurately predict the ranking of two given instances of control software–that is, the ability to predict which instance of control software performs better than the other in reality. To compute the *best*, we consider all 43520 possible pairwise comparisons of instances of control software within the individual studies from which we collected them so as to ensure fair comparisons. For each possible pair of instances {X,Y}, we compute the *best* as

2 $$best=\left\{\begin{array}{ll}1, & if\,argma{x}_{{\rm{X}}| {\rm{Y}}}\left({\bar{\theta }}_{{\rm{X}}},{\bar{\theta }}_{{\rm{Y}}}\right)=argma{x}_{{\rm{X}}| {\rm{Y}}}\left({\theta }_{{\rm{X}}},{\theta }_{{\rm{Y}}}\right),\\ 0, & otherwise.\end{array}\right.$$

where *θ*_{X|Y}_ is the expected performance observed in reality, and ${\bar{\theta }}_{\left\{{\rm{X}}| {\rm{Y}}\right\}}$ is the one estimated by a predictor. For a pair of instances of control software, the *best* is 1 if a predictor correctly infers the better performing one; 0 otherwise.

We report in Fig. 2b the mean *best* of each predictor over all possible pairs {X,Y} of instances of control software, which is to be maximized. Results show that evaluations on *M*_*A*_ lead to the correct best performing method for less than 50% of the possible pairs as it obtains a *best* of 0.47. The accuracy of the other predictors is slightly above 50%, with a value of 0.54 for ${P}_{{M}_{B}}$ and 0.51 for ${P}_{{R}_{1}}$. The improvement of ${P}_{{M}_{B}}$ over ${P}_{{M}_{A}}$ is of 14.4%, the one of ${P}_{{R}_{1}}$ is of 7.4%. As the lines in Fig. 2b representing the 95% confidence interval on the mean *best* do not overlap, the improvement of ${P}_{{M}_{B}}$ and ${P}_{{R}_{1}}$ over ${P}_{{M}_{A}}$ is significant, and ${P}_{{M}_{B}}$ is the most accurate predictor.

### Impact of wrong predictions: the regret

The quantity *regret* measures the loss incurred due to wrong predictions of the best performing instance of control software of a given pair {X,Y}. The *regret* is computed as the difference between the real performance of the best performing instance of control software and the real performance of the instance predicted to be the best performing one. To aggregate the results obtained across different missions, we normalize the difference with the maximal performance observed in reality. For each possible pair of instances of control software {X,Y}, we compute the relative *regret* as

3 $$regret=\left({\rm{\max }}({\theta }_{{\rm{X}}},{\theta }_{{\rm{Y}}})-{\theta }_{argma{x}_{{\rm{X}}| {\rm{Y}}}\left({\bar{\theta }}_{{\rm{X}}},{\bar{\theta }}_{{\rm{Y}}}\right)}\right)/{\rm{\max }}({\theta }_{{\rm{X}}},{\theta }_{{\rm{Y}}}),$$

where *θ*_{X|Y}_ is the performance observed in reality, and ${\bar{\theta }}_{\left\{{\rm{X}}| {\rm{Y}}\right\}}$ is the one estimated by a predictor. For a given pair of instances of control software, if a predictor correctly determines which instance yields the best performance in reality, the relative *regret* is 0. Otherwise, the relative *regret* takes a value between 0 and 1, and it is an indicator of the impact of making a mistake in predicting the best performing instance: the larger the difference of real-world performance of two instances of control software, the larger the *regret*, and vice versa. The relative *regret* is therefore to be minimized.

We report in Fig. 2c the mean *regret* of all predictors. Results show that ${P}_{{M}_{A}}$ obtains the largest *regret*, with a value of 0.26; ${P}_{{R}_{1}}$ has the second largest one with a value of 0.24, and ${P}_{{M}_{B}}$ obtains the smallest one with a value of 0.22. The improvement of ${P}_{{M}_{B}}$ and ${P}_{{R}_{1}}$ over ${P}_{{M}_{A}}$ is significant. It should be noted that this improvement of the pseudo-reality predictors over ${P}_{{M}_{A}}$ (and the one regarding the *best* reported in the previous subsection) is marginal with respect to the improvement we observed regarding the *error* (Fig. 2a). This difference of magnitude throughout the evaluation criteria shows the importance of analyzing the estimations of relative performance of pairs of instances of control software together with the accuracy of the performance estimations of instance individually: a predictor might poorly estimate performance of two instances, yet it might estimate the relative performance correctly, leading to the correct prediction of the best performing design method.

### Analysis based on the origin of the control software

Here we divide the data contained in DS 1 with respect to approach used to generate the instances of control software. We consider three families: the neuroevolutionary one regroups instances in the form of neural networks that have been generated by neuroevolutionary design methods^10^, the modular one regroups instances in the form of probabilistic finite-state machines or behavior trees that have been produced by modular design methods^4,33^, and the human one regroups instances produced by human designers. We analyze the accuracy of the predictors within and across these three families.

**Fig. 3:**
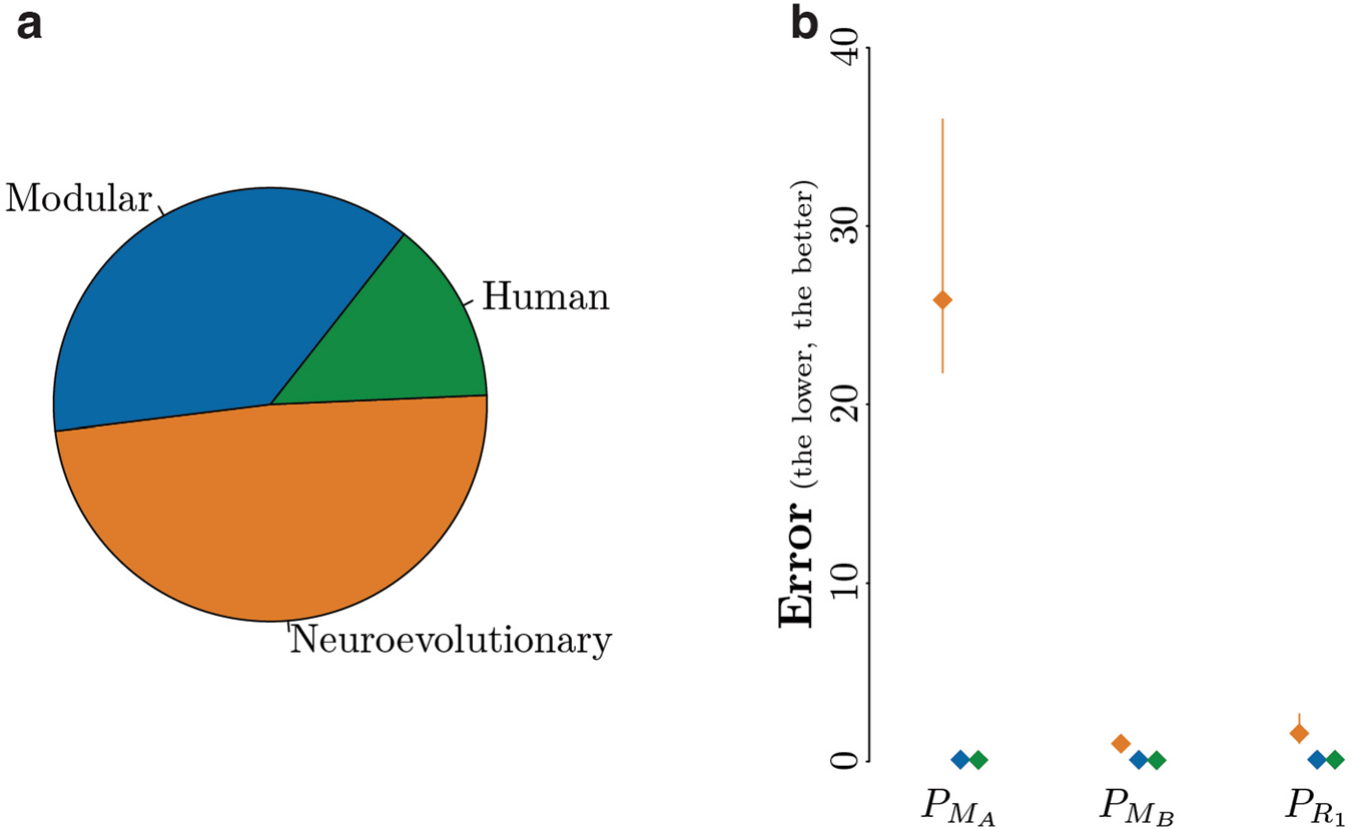
*Error* of the predictors for different families of design methods: neuroevolutionary methods, modular methods, and human designers. (**a**) The group ‘neuroevolutionary’ refers to instances of control software produced by neuroevolutionary methods, the group ‘modular’ refers to those produced by modular methods, and the group ‘human’ refers to those produced by human designers. (**b**) median *error*. The orange left-most points represent the median *error* when considering control software produced by neuroevolutionary robotics methods; the blue central points represent the one when considering control software produced by modular methods; the green right-most points represent the one when considering those produced by human designers. The vertical segments represent the 95% confidence interval.

Of the 1021 instances of control software considered, 49% have been produced by 10 methods belonging to the neuroevolutionary family, and 38% by 6 methods belonging to the modular family; the remaining instances have been created by human designers (Fig. 3a). Throughout the original studies from which we collected these instances of control software, methods belonging to the neuroevolutionary approach have shown to suffer a relatively large performance drop: they produced control software that performed well in simulation (that is, on the design model *M*_*A*_), but poorly in reality. On the other hand, the modular methods and human designers produced control software that performed satisfactorily in both simulation and reality. The analysis of the *error* yield by the predictors on the three families of methods confirms these observations (Fig. 3). In fact, the median *error* of ${P}_{{M}_{A}}$ is considerably higher for the control software produced by neuroevolutionary methods (almost 26) with respect to those produced by the modular methods (0.1) and human designers (0.08). A similar trend can be observed for pseudo-reality predictors ${P}_{{M}_{B}}$ and ${P}_{{R}_{1}}$ (Fig. 3b): the median *error* is substantially larger for performance predictions of neuroevolutionary instances (1 and 1.58, respectively) than for the one of modular instances (0.09 and 0.1) or for the one of the human instances (0.06 and 0.09). The pseudo-reality predictors are noticeably more accurate at predicting performance of the neuroevolutionary instances of control software than ${P}_{{M}_{A}}$, and ${P}_{{M}_{B}}$ is more accurate than ${P}_{{R}_{1}}$. The improvement of ${P}_{{M}_{B}}$ over ${P}_{{M}_{A}}$ and ${P}_{{R}_{1}}$ is significant with a confidence level of at least 95%. For what concerns the control software produced by modular methods and human designers, the difference in median *error* between ${P}_{{M}_{A}}$ and the pseudo-reality predictors is negligible.

**Fig. 4:**
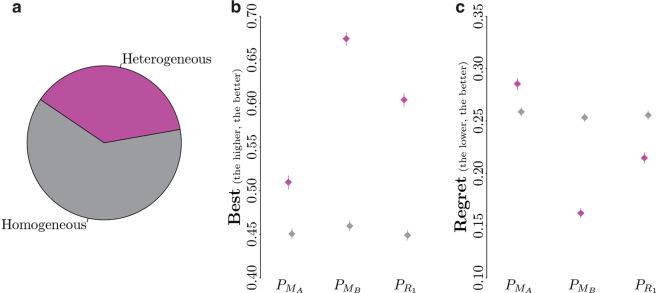
*Best* and *regret* of the predictors for different comparisons of design methods. (**a**) The group ‘homogeneous’ refers to comparisons of control software generated by design methods belonging to the same family: both methods belong to either the neuroevolutionary approach, the modular one, or have been designed by a human. The group ‘heterogeneous’ refers to comparisons of control software generated by methods of different families. (**b**) Mean *best*; (**c**) mean *regret*. In both plots, and for each predictor, the fuchsia left-most points represent the mean best or mean regret when considering heterogeneous comparisons; the gray right-most points represent the ones when considering homogeneous comparisons. The vertical segments represent the 95% confidence interval on the respective metric.

Among the 43520 possible comparisons of instances of control software available in DS 1, 62% are homogeneous comparisons—that is, comparisons of pairs of instances produced by design methods belonging to the same family—and 48% are heterogeneous comparisons—that is, the two instances have been produced by design methods belonging to different families. Because two instances belonging to the same family display similar ranges of performance in reality, predicting which of the two will perform better is difficult. On the other hand, it is less challenging for two instances conceived by design methods belonging to different families as their real-world performance differ noticeably. Figure 4 reports the *best* and *regret* of the predictors for comparisons of homogeneous and heterogeneous pairs of design methods. Surprisingly, the mean *best* of the pseudo-reality predictors ${P}_{{M}_{B}}$ and ${P}_{{R}_{1}}$ is only slightly higher (better) than the one of ${P}_{{M}_{A}}$ when considering homogeneous comparisons (Fig. 4b). In fact, ${P}_{{M}_{A}}$ obtains a mean *best* of 0.451, whereas ${P}_{{M}_{B}}$ and ${P}_{{R}_{1}}$ obtain one of 0.46 and 0.449, respectively. The three predictors thus fail to correctly predict which instance of control software performs better in reality for more than 50% of the comparisons. Also for the case of the mean *regret*, values are extremely close: ${P}_{{M}_{A}}$ obtained a mean *regret* of 0.259, whereas ${P}_{{M}_{B}}$ and ${P}_{{R}_{1}}$ both obtained one of 0.253 and 0.256, respectively (Fig. 4c).

Although all three predictors have higher mean *best* for heterogeneous pairs of instances of control software than for homogeneous ones, the difference is minor for what concerns ${P}_{{M}_{A}}$ in comparison with the ones of ${P}_{{M}_{B}}$ and ${P}_{{R}_{1}}$ (Fig. 4b). Whereas these last two predictors are able to correctly predict which instance is the best performing one in respectively 67.3% and 60.4% of the heterogeneous comparisons of DS 1, ${P}_{{M}_{A}}$ can only do so for 51%. As a result of the poor accuracy of ${P}_{{M}_{A}}$ in predicting the best performing instance of control software for heterogeneous pairs, its mean *regret* is considerably larger with respect to the one for homogeneous pairs. In fact, ${P}_{{M}_{A}}$ is the only predictor that has a larger mean *regret* for heterogeneous pairs than for homogeneous ones: the one of ${P}_{{M}_{B}}$ drops to 0.16, whereas the one of ${P}_{{R}_{1}}$ decreases to 0.21 (Fig. 4c).

An analysis of the *best* and *regret* of the predictor ${P}_{{M}_{A}}$ confirms the assumption that predicting the best performing instance of control software out of a heterogeneous pair is more straightforward as the two instances are more likely to display different ranges of performance in reality. In fact, although the *best* of ${P}_{{M}_{A}}$ is higher for heterogeneous comparisons than for homogeneous ones, its mean *regret* for heterogeneous pairs is also larger than for homogeneous ones. This is explained by the fact that the difference in real-world performance between instances of control software for a heterogeneous pair is important, leading to a larger relative *regret*—that is, a larger loss—when the wrong instance is predicted to be the best performing one in reality. On the contrary, mistakes in detecting the best performing instance in homogeneous pairs might be more frequent, but they result in a smaller loss as the performance in reality are more likely to be similar.

### Correlation between predictions and width of the pseudo-reality gap

The notion of width of a pseudo-reality gap refers to a measure of the difference between the design model in which control software is conceived and the pseudo-reality model in which it is evaluated^18^. In this section, we evaluate each instance of control software on 30 models sampled from the range *R*, and we study the correlation between the three evaluation criteria and the width of the pseudo-reality gap these models create with the design model. We compute (a) the ℓ^1^ norm of differences between two models and (b) their cosine similarity to quantify the extent to which the pseudo-reality models sampled from *R* differ from the design model *M*_*A*_. These two measures are described in the Methods section below. With this analysis, our goal is to learn whether the measures considered provide meaningful information, such as the possible existence of a subrange of *R* that leads to more accurate predictions. If it is the case, we envision that these measures (or similar ones) would be helpful for the future definition of new, better predictors.

**Fig. 5:**
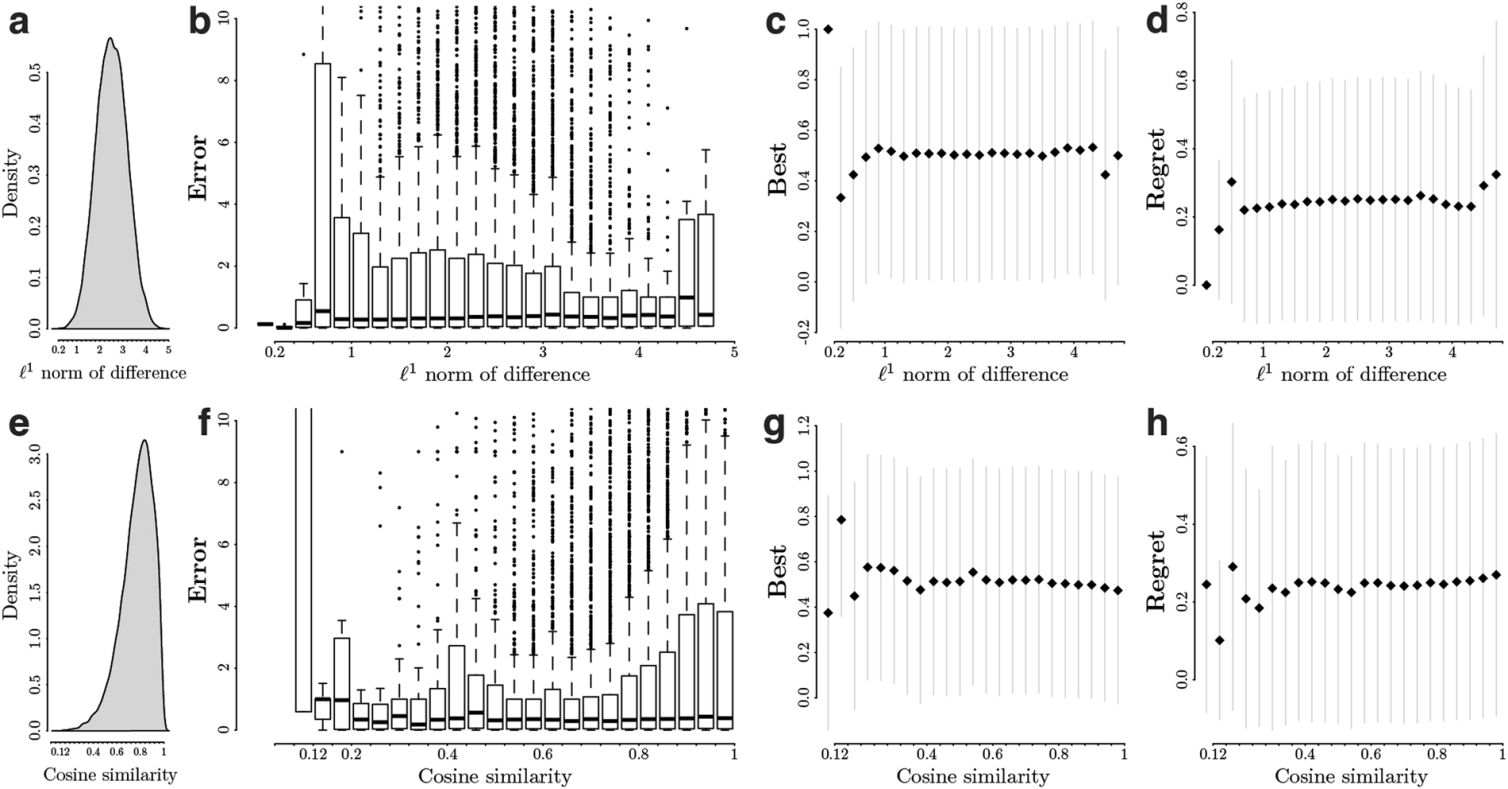
Width of pseudo-reality gap: *error*, *best*, and *regret*. For each instance of control software included in DS 1, and any possible pairwise combinations of these instances, 30 models were randomly sampled from the range *R*. We computed the width of the pseudo-reality gap created between the sampled models and the design model *M*_*A*_ using two measures: the ℓ^1^ norm of differences, and the cosine similarity. (**a**) Distribution of the width measured as the ℓ^1^ norm of the differences between the *R*_1_ models and *M*_*A*_, with mean equal to 2.5. (**b**), (**c**), and (**d**) *error*, *best*, and *regret* with respect to the ℓ^1^ of the differences between the sampled models and the design one. Their Pearson correlation coefficients are equal to −0.01, 0.002, 0.01, respectively. (**e**) Distribution of the width computed as the cosine similarity between the *R*_1_ models and *M*_*A*_, with mean equal to 0.77. (**f**), (**g**), and (**h**) *error*, *best*, and *regret* with respect to the cosine similarity between sampled models and the design one. Their Pearson correlation coefficients are equal to 0.03, −0.02, 0.02, respectively. It is worth noting that the ℓ^1^ norm of differences between *M*_*A*_ and *M*_*B*_ is equal to 1.59, whereas their cosine similarity is equal to 0.89.

In Fig. 5, we report the distribution of the widths computed with the two aforementioned measures. We also discretize the widths and report the *error*, *best*, and *regret* computed across all the available instances of control software. In particular, we represent the *error* with box-and-whiskers boxplots, and plot the mean *best* and mean *regret* together with the associated standard deviations. In Supplementary Figs. 1, 2, we report the correlation between the widths and the *error*, *best*, and *regret* according the different groups considered in the previous section—that is, we consider the *error* of control software produced by neuroevolutionary, modular, and human design methods; and the *best* and *regret* resulting from homogeneous and heterogeneous comparisons of control software.

When computed as the ℓ^1^ norms of the differences between the models *R*_1_ and *M*_*A*_, the width ranges from 0 to 5, with the larger the norm, the wider the gap between the two models (Fig. 5a–d). The distribution of the widths of the pseudo-reality gap is symmetric around the mean 2.5 (Fig. 5a). The *error* slightly decreases as the width increases, but increases for widths greater than 4.4 (Fig. 5b). This suggests that a larger difference between evaluation and design model yields better accuracy, but that a too larger difference might be counterproductive. In Supplementary Fig. 1, it can be observed that the *error* for control software produced by neuroevolutionary methods decreases when width increases, whereas the one for control software produced by modular methods remain stable. At visual inspection, the *error* for control software produced by human designers seems to increase as the width increases, but the Pearson correlation coefficient of −0.04 indicates a negative correlation. When considering all instances of control software, the *best* first increases, then quickly plateaus for widths larger than 1 (Fig. 5b). The same can be observed when only considering homogeneous comparisons of control software, whereas the *best* tends to keep increasing until widths larger that 4.4 (Supplementary Fig. 2a,b). For what concerns the *regret*, it remains relatively stable for all widths.

In a positive space such as the one considered here, the cosine similarity ranges from 0 to 1, with the lower the value, the wider the gap between the two models (Fig. 5e–h). When models from *R* are sampled uniformly like we did in this study, the distribution of the width is skewed to the left (Fig. 5e). Figure 5f shows that the *error* slightly increases as the design model and the evaluation ones get closer—in other words, when the gap reduces. Figure 5g-f show the *best* decreasing and the *regret* increasing as the gap gets smaller, respectively. Supplementary Fig. 1d–f show that, as design and evaluation models get closer, the *error* of the instances of control software produced by neuroevolutionary methods increases more decidedly than the one of those produced by modular methods or designed by humans. Supplementary Fig. 1e–h show that, for heterogeneous pairs of control software, the *best* decreases and the *regret* increases as the gaps narrow down, whereas it remains relatively constant when considering homogeneous pairs.

Overall, trends are more visible when one observes the cosine similarity than the ℓ^1^ norms of the differences. The Pearson correlation coefficients reported in the captions of Fig. 5 support this, with values slightly greater when using the cosine similarity, whereas those reported in Supplementary Fig. 1-2 exacerbate the differences between the different groups studied.

### Varying the sample size *k* of predictor *P*_*R*_

**Fig. 6:**
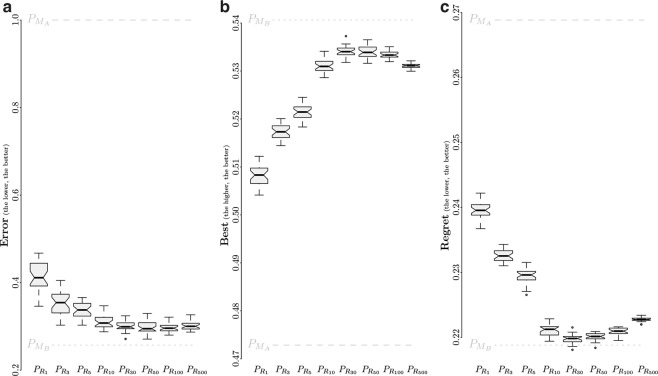
Effect of the sampling size on the predictor ${P}_{{R}_{n}}$. (**a**) median *error*, (**b**) mean *best*, and (**c**) mean *regret*. The results are presented using notched box-and-whiskers plots, where the notches represent the 95% confidence interval on the median. If notches on different boxes do not overlap, the medians of the corresponding predictors differ significantly with a confidence of at least 95%. Each box represent the metrics resulting from 30 executions of the predictors. Performance of ${P}_{{M}_{A}}$ (dotted line) and ${P}_{{M}_{B}}$ (dashed line) are added for comparison.

The predictor ${P}_{{R}_{k}}$ consists, for a given instance of control software, in its evaluation on *k* models sampled from the range *R* of models. The resulting prediction of the performance of the instance is the median of the estimated performance resulting from the *k* evaluations. We consider *k*∈{1,3,5,10,30,50,100,500} to study the effect of the sample size on the accuracy of the predictor. We apply ${P}_{{R}_{k}}$ on DS 1 30 times for each *k* considered, and present the resulting median *error*, mean *best*, and mean *regret* of the 30 executions in the form of box-and-whiskers plots (Fig. 6).

In Fig. 6a, each box represents 30 median *error*, each resulting from the execution of a predictor on DS 1. In addition to the diminution of the variance with the increase of the number of models sampled from *R* for the estimation of the expected performance of the instance of control software, the results show that the accuracy increases (i.e., the *error* decreases). The best score is obtained by ${P}_{{R}_{50}}$, with a median value of 0.295, which corresponds to an improvement of 28% over ${P}_{{R}_{1}}$. One can notice a slight increase of the *error* as the sample size exceeds 50 models (that is, for predictors ${P}_{{R}_{100}}$ and ${P}_{{R}_{500}}$). However, we did not detect significant differences between ${P}_{{R}_{10}}$, ${P}_{{R}_{30}}$, ${P}_{{R}_{50}}$, ${P}_{{R}_{100}}$ and ${P}_{{R}_{500}}$. In Fig. 6b, each box represents 30 mean *best*. The plot shows a clear improvement of the accuracy until the sample size reaches 30 models, the accuracy then plateaus. Surprisingly, the accuracy is significantly lower when 500 models are sampled (that is, ${P}_{{R}_{500}}$); the accuracy is then equivalent to estimating performance with 10 models, yet with lower variance. Both ${P}_{{R}_{30}}$ and ${P}_{{R}_{50}}$ obtain a score of 0.534, which is an improvement of about 5% with respect to the one of ${P}_{{R}_{1}}$. Figure 6b, in which each box represents 30 mean *regret*, shows similar trends, only inverted. In fact, one can see an improvement of the accuracy (i.e., a decrease of the *regret*) as the number of sampled models *n* increases. The *regret* is the lowest for ${P}_{{R}_{30}}$, with a value of 0.22 which correspond to an improvement of 8% over ${P}_{{R}_{1}}$, then increases for larger sample size. In fact, ${P}_{{R}_{500}}$ is significantly worst than ${P}_{{R}_{10}}$.

Overall, considering multiple models sampled from *R* to estimate the performance of an instance of control software leads to better accuracy. However, our results shows that there is an optimal value in the number of models, and that larger sample size does not necessarily means higher accuracy. Yet, even if very large sample sizes are suboptimal, they are still significantly more accurate than very small ones or than ${P}_{{M}_{A}}$.

## Discussion

We have shown that the pseudo-reality predictors considered in this study estimate more accurately the real-world performance of control software than the design model *M*_*A*_. This observation might lead one to assume that, for example, the pseudo-reality model *M*_*B*_ is a more truthful representation of reality than *M*_*A*_, and that automatically generating control software on the basis of *M*_*A*_ is a bad design choice. As mentioned in the Introduction, a number of approaches to handle the reality gap are motivated by the working hypothesis that the more accurate the simulations, the smoother the transition to reality^13,19–22^. Following this hypothesis, one could assume that designing control software on the basis of model *M*_*B*_ rather than on *M*_*A*_ would result in better performance in reality. We conducted an experiment to put this inference to the test.

**Fig. 7:**
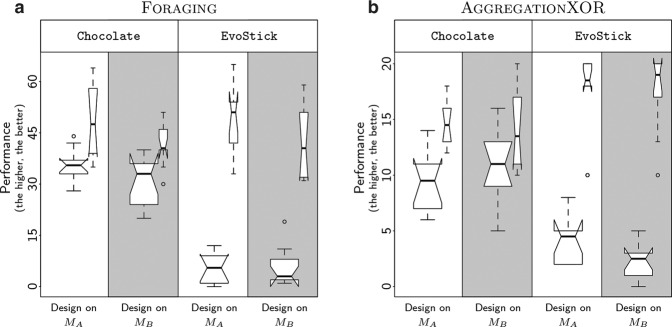
Performance of control software automatically designed on the basis of *M*_*A*_ and *M*_*B*_. The performance of EvoStick and Chocolate on (**a**) Foraging and (**b**) AggregationXOR, to be maximized, is represented by notched box-and-whiskers plots. Notches on the boxes represent the 95% confidence interval on the median, and allow for a convenient visual analysis of the results: if the notches of two boxes do not overlap, the difference between the two boxes is significant with a confidence of at least 95%^64^. White areas represent the performance of control software designed on the basis of model *M*_*A*_; gray areas represent the one of control software designed on model *M*_B_. Narrow boxes represent the performance of the control software obtained in simulation, that is, evaluated on the model used during the design; wide boxes represent the performance of the same control software in reality. Descriptions of the two missions and two design methods are given in Methods.

In this experiment, we generated control software using two previously proposed design methods—Chocolate and EvoStick—to solve two missions—AggregationXOR and Foraging. Details about these design methods and missions are available in Methods. We specifically chose to apply these two design methods on these two missions as this is the same experimental setup that was adopted to define the pseudo-reality predictors ${P}_{{M}_{B}}$^17^ and ${P}_{{R}_{1}}$^18^. We execute the two design methods on the two missions twice: once to generate control software on the basis of model *M*_*A*_, and once to do so on the basis of model *M*_*B*_. To account for stochasticity, we execute each design method 10 times for each model on each mission, resulting in the generation of a total of 80 instances of control software. We then evaluate each instance once on a swarm of 20 e-puck robots, and report the results in the form of box-and-whiskers plots (Fig. 7). Results reveal that designing control software on the basis of *M*_*B*_ does not yield better performance on the physical robots than designing them on the basis of *M*_*A*_. In fact, effects of the reality gap occur to the same degree regardless of the model used during the design: the control software designed by Chocolate suffer from mild performance drops in reality, whereas the one designed by EvoStick suffer from important ones (Table 1). These results indicate that *M*_*A*_, which has been used as design model for generating control software for robot swarms in several studies^15,16,27,32,33,36,37,41,42^, is not to be blamed for performance drops eventually experienced by some design methods. Rather, these results—together with those discussed in the previous section—substantiate the contention that the effects of the reality gap are due to the fact that design methods might overfit the model on the basis of which they operate, hence producing control software that is not robust to the differences of conditions experienced once ported on physical robots^43^.

**Table 1:** Performance drop experienced by Chocolate and EvoStick across the two missions considered, with the respective 95% confidence interval.

Method	Design model	Mean normalized performance drop
Chocolate	*M*_*A*_	0.29[0.20,0.38]
Chocolate	*M*_*B*_	0.25[0.13,0.37]
EvoStick	*M*_*A*_	0.81[0.74,0.88]
EvoStick	*M*_*B*_	0.86[0.80,0.92]

To aggregate across the missions, we normalized the performance drop with the performance obtained in simulation: they are computed as $\frac{{P}_{s}(i)-{P}_{r}({\rm{i}})}{{P}_{s}(i)}$ for each instance of control software *i*, where *P*_*s*_ and *P*_*r*_ are the performance in simulation and reality, respectively. A normalized performance drop of 0.25 implies that the performance in reality is 25% lower than the one obtained in simulation.

### Future research direction

Although the pseudo-reality predictors we considered are more accurate than the current practice—which consists in evaluating control software on the design model—they did not yield perfect estimations of real-world performance, and there is therefore room for improvement.

We foresee that one could define predictors with higher accuracy by following two parallel avenues. One might wish to consider a wider range of possible pseudo-realities by including offsets to the noise applied to the sensors and actuators of the robotic platform, and multiple distributions. One might also go beyond noise and consider different parameters, or consider different structures of the simulation model. Our study on the number of models sampled shows that the sampling size has a significant influence on the accuracy. Our study on the correlation between accuracy and differences between the design model and the sampled ones suggests that an optimal subrange of models (or a single model) that leads to higher performance exists. Rather than searching for an optimal subrange or a unique model by hand, like we did for *M*_*B*_, an alternative could be to define an automatic procedure instead. This procedure could use an optimization algorithm, one or several evaluation criteria to guide the search, and be based on the decomposition of the available control software into training and evaluation sets as it is typically done in machine learning^44,45^.

It is reasonable to assume that simulation predictors, however accurate they might become, will never replace experiments with physical robots. Yet, we foresee that they could considerably reduce the amount of tests with physical robots, and would therefore facilitate the research in off-line design of robot swarms. Comparisons between performance assessed on the design model and those yielded by an accurate predictor could be used as an indicator of the robustness of control software to the reality gap. With such indicator, preliminary simulation-only tests could be performed and used to validate ideas and design methods, or disregard those that do not seem promising—a couple of studies have already used the predictor ${P}_{{M}_{B}}$ for this purpose^41,46^. Similarly, simulation predictors could be used to implement early stopping mechanisms^47,48^ within design methods to halt the design process after the optimal number of steps, thus preventing overdesign^16^. Simulation predictors could also be adopted to prune the search space of possible candidate solutions, that is, remove those that are predicted not to be robust to the reality gap. Koos *et al*.^22^ used this idea in a method they called the transferability approach. In this method, a mission-specific model that predicts the robustness of control software to the reality gap is iteratively build with periodic tests on physical robots performed during the design. A mission-agnostic predictor like the ones we propose could (at least partially) replace robot tests and speed up the design process considerably.

## Methods

### Dataset DS 1

DS 1 contains the real-world performance of 1021 instances of control software generated by 18 different off-line design methods for 45 missions^31^. The majority of these instances have been evaluated once on physical robots, and a few have been evaluated multiple times under different initial configurations of the swarm—that is, positions and orientations of the robots. In total, DS 1 contains 1385 observations of real-world performance, each resulting from the conduction of a complete experiment. For each real-world evaluation, DS 1 also contains the predictions yield by ${P}_{{M}_{A}}$, ${P}_{{{\rm{M}}}_{B}}$, and of 1380 models uniformly sampled from the range *R* of possible pseudo-reality models–see subsection Predictors for more details. The predictions were obtained by executing the 1021 available instances of control software on the different simulation models and with the same initial configurations of the swarm that were used during the evaluations on the physical robots.

### Robotic platform

The e-puck is a small two-wheeled robot commonly used in swarm robotics^40^. All the control software collected in this study has been generated to be executed on e-pucks enhanced with additional hardware^49^: the Overo Gumstix, the ground sensor module, and the range-and-bearing module^50^. This e-puck version can detect obstacles and measure the ambient light, perceive the gray-level color of the floor situated under its body, and detect the number of neighboring peers situated in an approximate range of 0.70 m as well as estimate their relative position.

The capabilities of the robot are formally described in a reference model^51^ which serves as an interface for the control software: it describes what variables associated to the capabilities of the robots are accessible to the control software. The vast majorities of the design methods used to generate the control software collected have access to the reference model RM1.1. Two design methods, namely Gianduja and EvoCom, have access to reference model RM2, which enables the robots to send and react to a one bit message broadcasted via the range-and-bearing module. The two reference models are depicted in Table 2.

**Table 2:** Reference model RM1.1 and RM2^51^. The range-and-bearing vector $V={\sum }_{m=1}^{n}\,(\frac{1}{1+{r}_{m}},\angle {b}_{m})\,$, where *r*_*m*_ and ∠*b*_*m*_ are range and bearing of neighbor *m*, respectively, points to the aggregate position of the neighboring peers. If *n* = 0, then *V* = (1, ∠0). The vector *V*_*b*_ is computed as *V* by restricting to the *b* broadcasting neighboring robots. The variables are updated every 100 ms.

Sensor	Variables	RM1.1	RM2
proximity	*prox*_*i*_ ∈ [0, 1], with *i* ∈ {0, 1, ..., 7}	✗	✗
light	*light*_*i*_ ∈ [0, 1], with *i* ∈ {0, 1, ..., 7}	✗	✗
ground	*ground*_*i*_ ∈ {*white, gray, black*}, with *i* ∈ {0, 1, 2}	✗	✗
range-and-bearing	*n* ∈{0, 1, ..., 19}	✗	✗
	*V* ∈([0.5, 20], [0, 2*π*] rad)	✗	✗
	*b* ∈{0, 1, ..., 19}		✗
	*V*_*b*_ ∈ ([0.5, 20], [0, 2*π*] rad)		✗
**Actuator**	**Variables**	**RM1.1**	**RM2**
wheels	*v*_*l*_, *v*_*r*_ ∈ [−0.12, 0.12]ms^−1^	✗	✗
broadcast	*s* ∈ {*on, off*}		✗

### Predictors

The predictor ${P}_{{M}_{A}}$ consists in the execution of control software on the model used during its design. We call this model *M*_*A*_. *M*_*A*_ was originally conceived by Francesca *et al*.^15^. Noise is injected in the sensors and actuators of the robots. In the ARGoS3 simulator^39^, a uniform white noise is applied to the readings of the proximity, light, and ground sensors. A parameter *p*_*u*_ controls the level of noise: at every control cycle, for each sensor, a real value in the range $\left[-{p}_{u},{p}_{u}\right]$ is uniformly sampled and added to the reading. A Gaussian white noise is applied to the velocities of each wheel and parameter *p*_*g*_ controls the level of noise: at every control cycle, for each wheel, a value is sampled according to a Gaussian distribution with mean 0 and standard deviation *p*_*g*_, and added to the velocity. Finally, for the range-and-bearing module, a robot fails to estimate the relative position of a neighboring peer with probability *p*_*fail*_. In *M*_*A*_, the values of these parameters controlling the noise was determined following the best practice in automatic design^13,19^.

The predictor ${P}_{{M}_{B}}$ consists in the execution of control software on the pseudo-reality model *M*_*B*_ we defined via trial-and-error in a previous publication^17^. In that previous study, our goal was to find a pseudo-reality model *M*_*B*_ such that, when generating control software on the basis of *M*_*A*_ and evaluating it on *M*_*B*_, effects of the (pseudo-)reality gap similar to those observed Francesca *et al*.^15^ by would be reproduced. In their experiments, Francesca *et al*. compared the performance of control software generated by the design methods EvoStick and Vanilla to solve the missions AggregationXOR and Foraging (see below for descriptions). The authors generated the control software on the basis of *M*_*A*_ and ported it to a swarm of 20 e-puck robots. They observed a rank inversion between the two methods: in simulation, EvoStick outperformed Vanilla; but the other way around on the physical robots. To find a pseudo-reality model that would lead to similar results, we adopted a minimalist approach: we considered the simplest possible differences between two models, and stopped our search as soon as we found a convenient model. The difference between the model *M*_*B*_ and the design model *M*_*A*_ lies in the amount of noise applied to the actuators and sensors of the robots—see Table 3 for the values of these parameters. The differences of noise are, within the ARGoS3 simulator, the simplest discrepancies between two models one could possibly generate as the noise parameters explained above are loaded at run time from experimental files. Other pseudo-reality models that incorporate other types of differences with respect to *M*_*A*_ could probably lead to similar results, but they would require a modification of the ARGoS3 simulator itself.

**Table 3:** The models *M*_*A*_ and *M*_*B*_, and the ranges of possible values for models within the range *R*.

Actuator/Sensor	Parameter	*M*_*A*_	*M*_*B*_	Range *R*
wheels	*p*_*g*_	0.05	0.15	[0.00,0.20]
proximity	*p*_*u*_	0.05	0.05	[0.00,0.10]
light	*p*_*u*_	0.05	0.90	[0.00,1.50]
ground	*p*_*u*_	0.05	0.05	[0.00,0.10]
range-and-bearing	*p*_*fail*_	0.85	0.90	[0.70,1.00]

The values correspond the parameters of ARGoS3 controlling the noise applied to the actuator values and sensor readings.

The family of predictors ${P}_{{R}_{k}}$ consists in the evaluation of each instance of control software on *k* randomly sampled models taken from the range *R*. For an instance *i* of control software, *k* models are sampled from *R*, and *i* is evaluated *k* times—once for every *k* models, and the resulting median performance is taken as the predicted performance of *i*. Note that all *k* evaluations of *i* are performed using the same initial positions and orientations of the swarm. The range *R* and ${P}_{{R}_{1}}$ were previously defined^18^ and following the same procedure as for *M*_*B*_. We used here ${P}_{{R}_{k}}$ with *k*∈{1,3,5,10,30,50,100,500}. In practice, we implemented the predictors ${P}_{{R}_{k}}$ to sample, for each instance of control software, *k* of the 1380 performance predictions yielded by models sampled from *R* that are available in DS 1. To account for the stochasticity involved in the predictors ${P}_{{R}_{k}}$, we executed each of these predictors 30 times, and the results displayed in Figs. 2, 3, 4, 6 are aggregated results across all 30 executions. For example, the median error of ${P}_{{R}_{1}}$ reported in Fig. 2a is the average of 30 median errors, each median error resulting from one execution of ${P}_{{R}_{1}}$ on the instances of control software of DS 1, whereas the vertical lines represent the average 95% confidence interval of the 30 executions.

### Width of the pseudo-reality gap

Models considered in this paper differ from one another only in the value of 5 parameters controlling the noise applied to sensors and actuator of the robotic platform—see Methods for the details. Each model can therefore be identified by a vector in a five-dimensional space. We use the notation **v** to refer to the five-dimensional vector associated to a given model and the notation *v*_*p*_ with *p*∈{1,2,...,5} to refer to each of its 5 parameters. We compute (a) the ℓ^1^ norm of differences between two models and (b) their cosine similarity to quantify the extent to which a pseudo-reality model sampled from *R* differs from the design model *M*_*A*_.

The ℓ^1^ norm of differences between two vectors **a** and **b** is computed as

4 $${\ell }^{1}={\left\Vert {\bf{a}}-{\bf{b}}\right\Vert }_{1}=\mathop{\sum }\limits_{p=1}^{5}\left|{a}_{p}-{b}_{p}\right|.$$

The cosine similarity measures how similar the two vectors are, and is computed as

5 $$\frac{{\sum }_{p=1}^{5}{a}_{p}{b}_{p}}{\sqrt{{\sum }_{p=1}^{5}{a}_{p}^{2}}\sqrt{{\sum }_{p=1}^{5}{b}_{p}^{2}}}.$$

We consider the normalized version of the aforementioned measures. We normalize each term *v*_*p*_ of a vector **v** with respect to the lower *L*_*p*_ and upper *U*_*p*_ bounds of the range *R* given in Table 3. In particular, when a vector **d** represents a difference **a**-**b**, it is normalized into $\bar{{\bf{d}}}$ where

6 $${\bar{d}}_{p}=\left(\begin{array}{ll}\frac{{a}_{p}-{b}_{p}}{{U}_{p}-{b}_{p}}, & {\rm{if}}\,{a}_{p} > ={b}_{p};\\ \frac{{a}_{p}-{b}_{p}}{{b}_{p}-{L}_{p}}, & {\rm{if}}\,{a}_{p} < {b}_{p};\end{array}\right.{\rm{for}}\,p\,\in \{1,...,5\}.$$

Each component of $\bar{{\bf{d}}}$ ranges therefore between −1 and 1.

When a vector ${\bf{m}}=\{{m}_{1},...,{m}_{5}\}$ represents a model, it is normalized into a vector $\bar{{\bf{m}}}=\{{\bar{m}}_{1},...,{\bar{m}}_{5}\}$ where

7 $${\bar{m}}_{p}=\frac{{m}_{p}-{L}_{p}}{{U}_{p}-{L}_{p}}{\rm{for}}\,p\,\in \{1,...,5\}.$$

Each component of $\bar{{\bf{m}}}$ ranges therefore between 0 and 1.

### Design methods

We briefly describe the main characteristics of the design methods that produced the control software whose real-performance and estimated ones compose DS 1^38^. We divide these methods according to three families of approaches: the neuroevolutionary one, the modular one, and human one. A summary is given in Table 4. We refer the reader for the original papers for further details on these design methods.

**Table 4:** Summary of the design methods.

Method	Family	Control software architecture	Optimization algorithm	Reference model
Arlequin	Modular	PFSM	irace^58–60^	RM1.1
Chocolate	Modular	PFSM	irace	RM1.1
CMA-ES-mlp	Evolutionary	MLP	CMA-ES^53^	RM1.1
CMA-ES-slp	Evolutionary	SLP	CMA-ES	RM1.1
Coconut	Modular	PFSM	irace	RM1.1
C-Human	Human	PFSM	∅	RM1.1
EvoCom	Evolutionary	SLP	EA	RM2
EvoStick	Evolutionary	SLP	EA	RM1.1
Gianduja	Modular	PFSM	irace	RM2
Maple	Modular	BT	irace	RM1.1
NEAT-A-nl	Evolutionary	NN	NEAT^55^	RM1.1
NEAT-A-slp	Evolutionary	NN	NEAT	RM1.1
NEAT-B-nl	Evolutionary	NN	NEAT	RM1.1
NEAT-B-slp	Evolutionary	NN	NEAT	RM1.1
RandomWalk	Human	∅	∅	RM1.1
U-Human	Human	unconstrained	∅	RM1.1
Vanilla	Modular	PFSM	F-race^56,57^	RM1.1
xNES-mlp	Evolutionary	MLP	xNES^54^	RM1.1
xNES-slp	Evolutionary	MLP	xNES	RM1.1

PFSM stands for probabilistic finite state machines, BT for behavior trees, MLP for multi layer perceptron, SLP for single layer perceptron, EA for evolutionary algorithm. NN stands for neural network for which the topology is evolved and thus varies for each design.

#### Neuroevolutionary methods

Neuroevolutionary robotics is the most popular optimization-based approach for designing control software for robot swarms. In this approach, a neural network controls the individual robots: sensor readings are fed to the neural network as inputs, and the network’s output dictates the robot actuator values. The configuration of the neural network is optimized by an evolutionary algorithm.

With the exception of EvoCom, all implementations of the neuroevolutionary approach described here generate neural networks defined on the basis of reference model RM1.1. The neural networks produced have 25 input nodes, 2 output nodes, and the synaptic weights range in [−5, 5]. The 25 input nodes are organized as follows: 8 are dedicated to the readings of the proximity sensors, 8 to those of the light sensors, 3 to those of the ground sensors, 4 to the projections of the range-and-bearing vector V on four unit vectors that point to 45°, 135°, 225°, and 315°, 1 to the number of peers perceived, and 1 is a bias. The 2 output nodes define the velocity of the wheels.

The instances of control software considered in the dataset DS 1 have been generated by the following neuroevolutionary methods: **EvoStick** is a simple implementation of the neuroevolutionary robotics approached introduced by Francesca *et al*.^52^. It generates fully-connected, feed-forward neural networks that do not comprise hidden layers. EvoStick uses an evolutionary algorithm that has a population size of 100 individuals, evaluates each individual 10 times per generation, and produces novel populations based on elitism and mutation: the 20 best individuals are passed unchanged to the following generation, and the remaining 80 individuals are obtained via mutations applied to the same 20 best individuals. **CMA-ES-slp** is based on the evolutionary algorithm CMA-ES^53^. In CMA-ES, the population is described in statistical terms via the covariance matrix of its distribution. CMA-ES-slp adopts the same network topology as EvoStick. **CMA-ES-mlp** differs from CMA-ES-slp in the topology of the neural networks produced: the ones of CMA-ES-mlp contain one hidden layer composed of 14 nodes, including 1 bias node. **xNES-slp** is based on the evolutionary algorithm xNES^54^. xNES is identical to CMA-ES with the exception that its update rule is defined in a principled way. xNES-slp adopts the same network topology as EvoStick. **xNES-mlp** differs from xNES-slp only in the network topology adopted: it generates neural networks that contain a hidden layer of 14 nodes. **NEAT-A-slp** is based on the evolutionary algorithm NEAT^55^. With NEAT, both the synaptic weights and the topology of the neural networks are optimized. The initial population is composed of fully-connected, feed-forward neural networks that do not comport hidden layers. **NEAT-A-nl** differs from NEAT-A-slp only in the topology of the neural networks that compose the initial population. Here, the input nodes are initially not connected to the output nodes. **NEAT-B-slp** differs from NEAT-A-slp only in the value of some hyper-parameters of NEAT. Here, NEAT is configured so that it has a higher compatibility coefficient, does not penalize old species, and can generate recurrent neural networks. **NEAT-B-nl** differs from NEAT-B-slp only in the topology of the neural networks that compose the initial population: the input nodes are initially disconnected from the output nodes. **EvoCom** is derived from EvoStick and differs from all the previous methods in the input and output nodes that comport the neural networks it produces. Indeed, EvoCom is defined on the basis of RM2 which adds communication capabilities with respect to RM1.1. EvoCom generates neural networks that have 5 additional input nodes and 1 additional output node with respect to those of EvoStick. The 5 additional input nodes are dedicated to the detection of peers that are broadcasting a message: 1 is dedicated to the number of broadcasting peers perceived, and 4 to the projections of the range-and-bearing vector *V*_*b*_ on four unit vectors that point to 45°, 135°, 225°, and 315°.

#### Modular methods

The modular methods that produced control software comprised in the dataset DS 1 all belong to the AutoMoDe approach^15^. All these design methods generate control software by selecting and combining pre-defined modules: low-level behaviors that are executed by the robots, and conditions that are used to transition from one low-level behavior to another. With the exception of those of Gianduja, these modules are defined on the basis of reference model RM1.1.

Instances of control software considered in the dataset DS 1 have been generated by the following AutoMoDe methods: **Vanilla** is the first implementation of AutoMoDe^15^. Vanilla generates control software in the form of probabilistic finite-state machines that can comprise up to four states and up to four outgoing edges per state. Vanilla has its disposal a set of 12 software modules to conceive the probabilistic finite-state machines: 6 are low-level behaviors that are used as states, and 6 are conditions that are used as edges to transition from one behavior to another. All these software modules have been conceived by hand once-and-for-all in a mission-agnostic way by a human designer; some of them have parameters that are tuned during the design by the optimization algorithm to adjust their functioning. Vanilla uses the F-race optimization algorithm to select, tune, and combine the modules^56,57^. **Chocolate** is the second implementation of AutoMoDe, and only differs from Vanilla in the optimization algorithm adopted: Chocolate uses Iterated F-race (irace)^58–60^, an improved version of the F-race algorithm adopted by Vanilla. Chocolate has been introduced in^32^ and later used in^27,36,37,41,42,61^. **Maple** differs from Chocolate in the architecture of the control software produced: Maple selects, tunes, and combines the modules into behaviors trees^33,62^. **Arlequin** differs from Chocolate in the nature of the 6 pre-defined low-level behaviors that are at its disposal for conceiving control software: rather than combining manufactured behaviors, Arlequin combines behaviors that are automatically generated via the neuroevolutionary method EvoStick^36^. **Coconut** differs from Chocolate only in the number of pre-defined low-level behaviors that it can select, tune, and combine: Coconut embeds 2 additional exploration schemes within its modules^42^. **Gianduja** is derived from Chocolate differs from all the previous methods in the robot capabilities it exploits: Gianduja ’s modules are defined on the basis of reference model RM2, which extends RM1.1 with communication capabilities^34,41^. Gianduja operates on 8 low-level behaviors: 6 are the same as Chocolate extended with a binary parameter deciding whether a one bit message is broadcast while the behavior is performed, the 2 others make the robot go towards broadcasting peers, or in the opposite direction. Gianduja also operates on 8 conditions: 6 are the same as Chocolate, the 2 others are related to the number of broadcasting peers perceived.

#### Manual methods

In Francesca *et al*.^32^, the authors compared the performance of the control software generated by Vanilla, EvoStick, and Chocolate with the one conceived by 5 human experts in swarm robotics. Each expert had to solve two different missions, once following two different guidelines (that is, using two different manual design method). In both cases, the experts had to conceive control software on the basis of RM1.1. The two manual methods that have produced instances of control software present in the DS 1 are the following: **U-Human**, short for unconstrained-human, consists in letting the human designer implement the control software in the way they deem most appropriate, without any kind of restriction. **C-Human** short for constrained-human, consists in constraining the human designer to use the same software modules used by Vanilla and Chocolate. They are indeed constrained to combine the software modules into probabilistic finite-states machines that comport the same restrictions as those produced by those methods: up to four states with up to four outgoing edges.

In Hasselmann *et al*.^27^, the authors compared the performance of control software generated by 9 neuroevolutionary methods to the performance of a simple strategy consisting in the robots randomly roaming in the environment. This strategy was called **RandomWalk** in their paper, and we consider it as an instance of control software produced by manual method.

### Missions

**Fig. 8:**
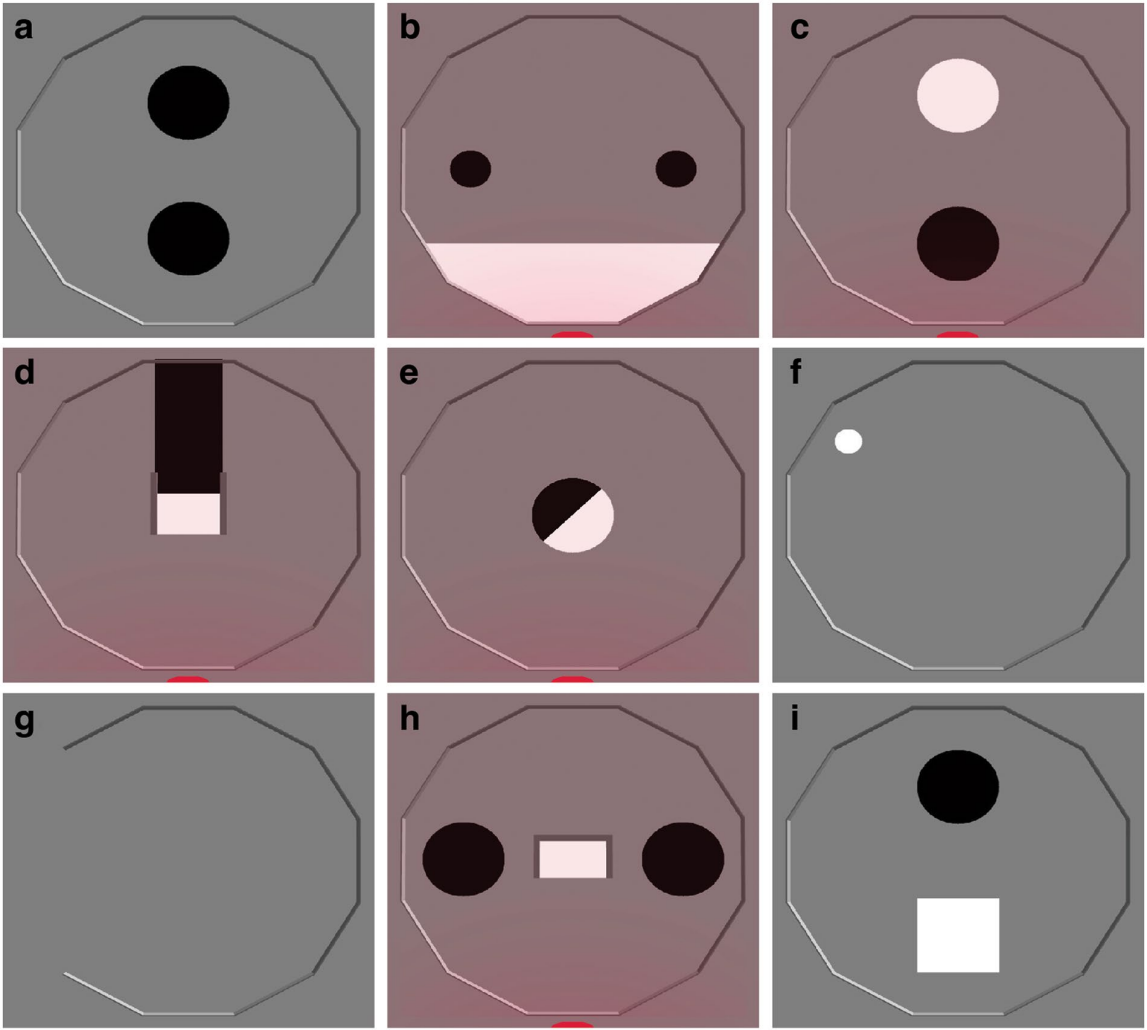
Illustrations of the arenas of some of the missions considered. (**a**) AggregationXOR. (**b**) Foraging. (**c**) Homing as studied by Francesca *et al*.^32^ under the name AAC. (**d**) DirectionalGate. (**e**) Decision. The circular area in the middle of the arena is either completely white or completely black. (**f**) Stop. (**g**) Unbounded arena. (**h**) Shelter. (**i**) SPC. The red glow in (**b**), (**c**), (**d**), (**e**), and (**h**) indicate the presence of a light source placed outside the south side of the arena that can be used by the robots to orient themselves.

We briefly describe here the missions to be solved by the control software collected. Some missions have been used in multiple studies, and might slightly different in some aspects. We refer the reader to the original papers for further information. Some missions have been studied with different types of objective functions which can be classified as *endtime*, if the performance is computed once at the very end of the experiment, and as *anytime*, if the performance is computed continuously throughout the experiment. Figure 8 illustrates some of the arenas corresponding to the missions described bellow.

In A**ggregationXOR**, the swarm must select one of the two black areas and aggregate there (Fig. 8a). The endtime objective function to be maximized is ${E}_{xor}={\rm{\max }}({N}_{l},{N}_{r})/N$, where *N*_*l*_ and *N*_*r*_ are the number of robots located on the left and right area, respectively; and *N* is the total number of robots. The objective function is maximized when all robots are either on the left or the right area. The anytime objective function to be maximized is ${A}_{xor}=\mathop{\sum }\limits_{t=1}^{T}{\rm{\max }}\left({N}_{l}(t),{N}_{r}(t)\right)/N$, where *T* is the duration of the mission. AggregationXOR has been studied in 3 of the works from which we collected data^27,37,61^. In **F**oraging, the swarm must retrieve virtual items from food sources and bring them the nest. The food sources are represented by small black disks, the nest is represented by a white area. A robot is deemed to pick up an item when it enters a food source, and drop the item as soon as it then enters the nest (Fig. 8b). A light source is placed behind the nest and can by used by the robots to orient themselves in the arena. The objective function to be maximized is *F*_*f*_=*I*, where *I* is the number of items retrieved. Foraging has been studied in 4 of the works from which we collected data^27,37,42,61^. In one of these works^42^, a variant of the mission has been studied, which is characterized by an unbounded arena (Fig. 8g). In **Homing**, the swarm must aggregate on an area designated as their home. The endtime objective function to be maximized is *F*_*h*_ = *N*_*home*_, where *N*_*home*_ is the number of robots located on the aggregation area. The anytime objective function to be maximized is ${F}_{h}=\mathop{\sum }\limits_{t=1}^{T}{N}_{home}(t)$, where *T* is the duration of the mission. This mission has been studied in different forms across the studies considered. In one of them^32^, the mission is called AAC—short for aggregation with ambient cues—and is characterized by the presence of two aggregation areas of different colors and a source of light indicating to the robots on which area to aggregate (Fig. 8c). In a second one^34^, the mission is called Aggregation: also in this case, the arena contains two aggregation areas of different colors, but not light source. In a third one^27^, the mission is called Homing: in this case, the arena contains only one aggregation area. In a fourth one^42^, the mission is called Aggregation: the arena contains only one aggregation area. The mission is studied twice: once in a closed arena and once with in an unbounded one (see Fig. 8g). In **DirectionalGate**, the swarm must traverse a gate from North to South^27^ (see Fig. 8d). The gate is positioned in the center of the arena and is identified by white floor. A light source is placed outside of the arena and in the axis of the gate to help the robots orientate themselves. A black corridor leads to the North entrance of the gate. The objective function, to be maximized, is ${F}_{DG}=K-\bar{K}$, where *K* is the number of times the robots traversed the gate in the right direction (North to South), and $\bar{K}$ the number of times they traversed it in the wrong direction (South to North). In **Decision**, the swarm must aggregate on the right-hand side or the left-hand side of the arena depending on the color of a circular area positioned in the middle of the arena^41^ (Fig. 8e). In each experimental run, the circular area can be either black or white, with equal probability. A light source is placed outside the arena, at its right. The objective function to be maximized is ${F}_{d}=24000-\mathop{\sum }\limits_{t=1}^{T}\mathop{\sum }\limits_{i=1}^{N}{I}_{i}(t)$, where *T* is the duration of the experiment, *N* is the number of robots, and ${I}_{i}(t)=0$ if robot *i* is in the correct half of the arena and ${I}_{i}(t)=1$ otherwise. In **Stop**, the swarm must find a small circular white spot as soon as possible, and stop right after (Fig. 8f). The objective function to be maximized is ${F}_{s}=48000-\,(\bar{t}N+\mathop{\sum }\limits_{t=1}^{\bar{t}}\mathop{\sum }\limits_{i=1}^{N}{\bar{I}}_{i}(t)+\mathop{\sum }\limits_{t=\bar{t}+1}^{T}\mathop{\sum }\limits_{i=1}^{N}{I}_{i}(t))\,$, where $\bar{t}$ is the time at which the first robot finds the white spot, *T* is the duration of the experiment, *N* is the number of robots, ${I}_{i}(t)=1$ if robot *i* is moving and ${I}_{i}(t)=0$ otherwise, and ${\bar{I}}_{i}(t)=1-{I}_{i}(t)$. In **GridExploration**, the swarm must explore the an arena and cover as much space as possible^42^. To measure the performance of the swarm, the arena is divided in a grid of 10 tiles by 10 tiles. For each tile, a counter *c* retains the time *t* elapsed since the last time the tile was visited by a robot. The counter is reset to 0 when a robot visits a tile. The objective function to be maximized is ${F}_{ge}=\mathop{\sum }\limits_{t=1}^{T}(\frac{1}{{N}_{tiles}}{\sum }_{j=1}^{{N}_{tiles}}-{c}_{j}(t))$, where *T* is the duration of the experiment, *N*_*tiles*_ is the number of tiles in the arena, and ${c}_{j}(t)$ is the value of the counter associated to tile *j*. The mission is studied twice:^42^ once in a bounded arena, once in an unbounded one (Fig. 8g). In **CFA**—short for coverage with forbidden areas—the swarm must cover the arena, avoiding the forbidden areas denoted by the three circular black areas^32^. The objective function, to be minimized, is ${F}_{CFA}=E\left[d(T)\right]$, where *E*[*d*(*T*)] is the expected distance, at the end *T* of the experiment, between a generic point of the arena and the closest robot that is not in the forbidden areas. In **LCN**—short for largest covering network–the swarm must create a connected network that covers the largest area possible in an empty arena^32^. Each robot covers a circular area of 0.35 radius. Two robots are considered to be connected if they are separated by less than 0.35. The objective function to be maximized is ${F}_{LCN}={A}_{C}$, where *C* is the largest network of connected robots, and *A*_*C*_ is the area covered by *C*. In **Shelter**, the swarm must aggregate in a rectangular white area surrounded by three walls and positioned in the center of the arena (Fig. 8h). A light source is positioned outside the arena, in front of the open side of the shelter. The arena also features two black circular areas that do not have any predefined purpose/role in the definition of the mission: they are noise-features of the environment. The objective function to be maximized is ${F}_{sh}=\mathop{\sum }\limits_{t=1}^{T}N(t)$, where *T* is the duration of the experiment and *N*(*t*) is the number of robots in the shelter at time *t*. This mission has been studied in two of the works considered;^27,32^ in one of them^32^ it is studied under the name SCA–short for shelter with constrained access. In **SPC**–short for surface and perimeter coverage–the arena contains a square white area and a circular black area (Fig. 8i). The swarm must cover the area of the white square and aggregate on the perimeter of the black circle^32^. The objective function to be minimized is ${F}_{spc}=E\left[{d}_{a}(T)\right]/{c}_{a}+E\left[{d}_{p}(T)\right]/{c}_{p}$, where $E\left[{d}_{a}(T)\right]$ is the expected distance, at the end *T* of experiment, between a generic point in the square area and the closest robot in the square, and $E\left[{d}_{p}(T)\right]$ is the expected distance between a generic point on the circumference of the circular area and the closest robot that intersects the circumference. *c*_*a*_ and *c*_*p*_ are scaling factors fixed to 0.08 and 0.06, respectively. If no robot is on the surface of the square area and/or on the perimeter of the circular area, $E\left[{d}_{a}(T)\right]$ and/or $E\left[{d}_{p}(T)\right]$ are undefined and we thus assign an arbitrarily large value to *F*_*spc*_.

## Supplementary information

Supplementary Figures

## Data Availability

All 1385 observations of real-world performance and predictions yield by ${P}_{{M}_{A}}$, ${P}_{{M}_{B}}$, and by the 1380 models uniformly sampled from the range *R* of pseudo-reality models are available in a public repository^31^. The data related to the experiment described in the Discussion section is available in a separate public repository^63^. It includes the performance obtained in simulation and in reality, videos of the experimental runs conducted on the physical robots, instances of control software produced and associated source code to evaluate them.

## Code availability

The instances of control software that produced DS 1, as well as the source code necessary to execute them–that is, the design methods, the simulator and the dependencies, the configuration files of the missions, together with scripts to compile the sources, generate necessary files, and execute the instances of control software–are available in a public repository^38^

## References

[1] Sahin, E. Swarm robotics: from sources of inspiration to domains of application. In Sahin, E. & Spears, W. M. (eds.) *Swarm Robotics, SAB*, vol. 3342 of *LNCS*, 10–20, 10.1007/978-3-540-30552-1_2 (Springer, Berlin, Germany, 2004).

[2] Dorigo M, Birattari M (2007). Swarm intelligence. Scholarpedia.

[3] Brambilla M, Ferrante E, Birattari M, Dorigo M (2013). Swarm robotics: a review from the swarm engineering perspective. Swarm Intelligence.

[4] Francesca G, Birattari M (2016). Automatic design of robot swarms: achievements and challenges. Frontiers in Robotics and AI.

[5] Hamann, H. *Swarm robotics: a formal approach* (Springer, Cham, Switzerland, 2018).

[6] Birattari M (2019). Automatic off-line design of robot swarms: a manifesto. Frontiers in Robotics and AI.

[7] Dorigo M, Theraulaz G, Trianni V (2020). Reflections on the future of swarm robotics. Science Robotics.

[8] Dorigo M, Theraulaz G, Trianni V (2021). Swarm robotics: past, present, and future [point of view]. Proceedings of the IEEE.

[9] Birattari M, Ligot A, Hasselmann K (2020). Disentangling automatic and semi-automatic approaches to the optimization-based design of control software for robot swarms. Nature Machine Intelligence.

[10] Nolfi, S. & Floreano, D. *Evolutionary Robotics: The Biology, Intelligence, and Technology of Self-Organizing Machines*, first edn. A Bradford Book (MIT Press, Cambridge, MA, USA, 2000).

[11] Floreano, D., Husbands, P. & Nolfi, S. Evolutionary robotics. In Siciliano, B. & Khatib, O. (eds.) *Springer Handbook of Robotics*, Springer Handbooks, 1423–1451, 10.1007/978-3-540-30301-5_62. First edition (Springer, Berlin, Heidelberg, Germany, 2008).

[12] Brooks, R. A. Artificial life and real robots. In Varela, F. J. & Bourgine, P. (eds.) *Towards a Practice of Autonomous Systems. Proceedings of the First European Conference on Artificial Life*, 3–10 (MIT Press, Cambridge, MA, USA, 1992).

[13] Jakobi, N., Husbands, P. & Harvey, I. Noise and the reality gap: the use of simulation in evolutionary robotics. In Morán, F., Moreno, A., Merelo, J. J. & Chacón, P. (eds.) *Advances in Artificial Life: Third european conference on artificial life*, vol. 929 of *Lecture Notes in Artificial Intelligence*, 704–720, 10.1007/3-540-59496-5_337 (Springer, Berlin, Germany, 1995).

[14] Silva F, Duarte M, Correia L, Oliveira SM, Christensen AL (2016). Open issues in evolutionary robotics. Evolutionary Computation.

[15] Francesca G, Brambilla M, Brutschy A, Trianni V, Birattari M (2014). AutoMoDe: a novel approach to the automatic design of control software for robot swarms. Swarm Intelligence.

[16] Birattari, M. *et al*. (eds.) *Swarm Intelligence–ANTS*, vol. 9882 of *Lecture Notes in Computer Science*, 45–57, 10.1007/978-3-319-44427-7_13 (Springer, Cham, Switzerland, 2016).

[17] Ligot, A. & Birattari, M. On mimicking the effects of the reality gap with simulation-only experiments. In Dorigo, M. *et al*. (eds.) *Swarm Intelligence–ANTS*, vol. 11172 of *LNCS*, 109–122, 10.1007/978-3-030-00533-7_9 (Springer, Cham, Switzerland, 2018).

[18] Ligot, A. & Birattari, M. Simulation-only experiments to mimic the effects of the reality gap in the automatic design of robot swarms. *Swarm Intelligence* 1–24, 10.1007/s11721-019-00175-w (2019).

[19] Miglino O, Lund HH, Nolfi S (1995). Evolving mobile robots in simulated and real environments. Artificial Life.

[20] Bongard, J. C. & Lipson, H. Once more unto the breach: co-evolving a robot and its simulator. In Pollack, J. B., Bedau, M. A., Husbands, P., Watson, R. A. & Ikegami, T. (eds.) *Artificial Life IX: Proceedings of the Conference on the Simulation and Synthesis of Living Systems*, 57–62. A Bradford Book (MIT Press, Cambridge, MA, USA, 2004).

[21] Zagal JC, Ruiz-del Solar J, Vallejos P (2004). Back to reality: crossing the reality gap in evolutionary robotics. IFAC Proceedings Volumes.

[22] Koos S, Mouret J-B, Doncieux S (2013). The transferability approach: crossing the reality gap in evolutionary robotics. IEEE Transactions on Evolutionary Computation.

[23] Floreano, D. & Mondada, F. Evolution of plastic neurocontrollers for situated agents. In Maes, P., Matarić, M. J., Meyer, J.-A., Pollack, J. B. & Wilson, S. W. (eds.) *From Animals to Animats 4. Proceedings of the Fourth International Conference on Simulation of Adaptive Behavior, SAB*, 402–410, 10.7551/mitpress/3118.003.0049 (MIT Press, Cambridge, MA, USA, 1996).

[24] Jakobi N (1997). Evolutionary robotics and the radical envelope-of-noise hypothesis. Adaptive Behavior.

[25] Jakobi, N. *Minimal simulations for evolutionary robotics*. Ph.D. thesis, University of Sussex, Falmer, UK (1998).

[26] Boeing, A. & BrÃ¤unl, T. Leveraging multiple simulators for crossing the reality gap. In *Proceedings of the International Conference on Control, Automation, Robotics and Vision–ICARCV*, 1113–1119, 10.1109/ICARCV.2012.6485313 (IEEE, Piscataway, NJ, USA, 2012).

[27] Hasselmann K, Ligot A, Ruddick J, Birattari M (2021). Empirical assessment and comparison of neuro-evolutionary methods for the automatic off-line design of robot swarms. Nature Communications.

[28] Quinn M, Smith L, Mayley G, Husbands P (2003). Evolving controllers for a homogeneous system of physical robots: structured cooperation with minimal sensors. Philosophical Transactions of the Royal Society of London. Series A: Mathematical, Physical and Engineering Sciences.

[29] Baldassarre G (2007). Self-organized coordinated motion in groups of physically connected robots. IEEE Transactions on Systems, Man, and Cybernetics, Part B (Cybernetics).

[30] Jones, S. *et al*. (eds.) *Distributed Autonomous Robotic Systems (DARS)*, vol. 6 of *SPAR*, 487–501, 10.1007/978-3-319-73008-0_34 (Springer, Cham, Switzerland, 2018).

[31] Ligot A, Birattari M (2022). Zenodo.

[32] Francesca G (2015). AutoMoDe-Chocolate: automatic design of control software for robot swarms. Swarm Intelligence.

[33] Kuckling, J. *et al*. (eds.) *Swarm Intelligence–ANTS*, vol. 11172 of *LNCS*, 30–43, 10.1007/978-3-030-00533-7_3 (Springer, Cham, Switzerland, 2018).

[34] Hasselmann, K. *et al*. (eds.) *Swarm Intelli**gence–ANTS*, vol. 11172 of *LNCS*, 16–29, 10.1007/978-3-030-00533-7_2 (Springer, Cham, Switzerland, 2018).

[35] Spaey, G. *et al*. Comparison of different exploration schemes in the automatic modular design of robot swarms. In Beuls, K. et al. (eds.) *Proceedings of the Reference AI & ML Conference for Belgium, Netherlands & Luxemburg, BNAIC/BENELEARN 2019*, vol. 2491 of *CEUR Workshop Proceedings* (CEUR-WS.org, Aachen, Germany, 2019).

[36] Ligot, A. *et al*. AutoMoDe-Arlequin: neural networks as behavioral modules for the automatic design of probabilistic finite state machines. In Dorigo, M. et al. (eds.) *Swarm Intelligence–ANTS*, vol. 12421 of *LNCS*, 271–281, 10.1007/978-3-030-60376-2_21 (Springer, Cham, Switzerland, 2020).

[37] Ligot A, Cotorruelo A, Garone E, Birattari M (2022). Towards an empirical practice in off-line fully-automatic design of robot swarms. IEEE Transactions on Evolutionary Computation.

[38] Ligot A, Birattari M (2022). Zenodo.

[39] Pinciroli C (2012). ARGoS: a modular, parallel, multi-engine simulator for multi-robot systems. Swarm Intelligence.

[40] Mondada, F. *et al*. The e-puck, a robot designed for education in engineering. In Gonçalves, P., Torres, P. & Alves, C. (eds.) *Proceedings of the 9th Conference on Autonomous Robot Systems and Competitions*, 59–65 (Instituto Politécnico de Castelo Branco, Castelo Branco, Portugal, 2009).

[41] Hasselmann K, Birattari M (2020). Modular automatic design of collective behaviors for robots endowed with local communication capabilities. PeerJ Computer Science.

[42] Spaey, G. *et al*. (eds.) *Artificial Intelligence and Machine Learning: BNAIC 2019, BENELEARN 2019*, vol. 1196 of *CCIS*, 18–33, 10.1007/978-3-030-65154-1_2 (Springer, Cham, Switzerland, 2020).

[43] Birattari, M., Ligot, A. & Francesca, G. Automode: a modular approach to the automatic off-line design and fine-tuning of control software for robot swarms. In Pillay, N. & Qu, R. (eds.) *Automated Design of Machine Learning and Search Algorithms*, Natural Computing Series, 10.1007/978-3-030-72069-8_5 (Springer, Cham, Switzerland, 2021).

[44] Hastie, T., Tibshirani, R. & Friedman, J. *The Elements of Statistical Learning: Data mining, Inference and Prediction*, second edn (Springer, Berlin, Germany, 2009).

[45] Goodfellow, I., Bengio, Y. & Courville, A. *Deep Learning*, first edn (MIT Press, Cambridge, MA, USA, 2016).

[46] Kuckling, J., van Pelt, V. & Birattari, M. Automatic modular design of behavior trees for robot swarms with communication capabilities. In Castillo, P. A. & Jiménez Laredo, J. L. (eds.) *Applications of Evolutionary Computation: 24th International Conference, EvoApplications 2021*, vol. 12694 of *Lecture Notes in Computer Science*, 130–145, 10.1007/978-3-030-72699-7_9 (Springer, Cham, Switzerland, 2021).

[47] Morgan, N. & Bourlard, H. Generalization and parameter estimation in feedforward nets: some experiments. In Touretzky, D. S. (ed.) *Advances in Neural Information Processing Systems 2, NIPS 1990*, 630–637 (Morgan Kaufmann Publishers, San Francisco, CA, USA, 1990).

[48] Caruana, R., Lawrence, S. & Giles, C. L. Overfitting in neural nets: backpropagation, conjugate gradient, and early stopping. In Leen, T. K., Dietterich, T. G. & Tresp, V. (eds.) *Advances in Neural Information Processing Systems 13*, 402–408 (MIT Press, Cambridge, MA, USA, 2001).

[49] Garattoni, L., Francesca, G., Brutschy, A., Pinciroli, C. & Birattari, M. Software infrastructure for e-puck (and TAM). Tech. Rep. TR/IRIDIA/2015-004, IRIDIA, Université libre de Bruxelles, Belgium (2015).

[50] Gutiérrez, Á. *et al*. Open e-puck range & bearing miniaturized board for local communication in swarm robotics. In Kosuge, K. (ed.) *IEEE International Conference on Robotics and Automation, ICRA*, 3111–3116, 10.1109/ROBOT.2009.5152456 (IEEE, Piscataway, NJ, USA, 2009).

[51] Hasselmann, K. *et al*. Reference models for AutoMoDe. Tech. Rep. TR/IRIDIA/2018-002, IRIDIA, Université libre de Bruxelles, Belgium (2018).

[52] Francesca, G., Brambilla, M., Trianni, V., Dorigo, M. & Birattari, M. Analysing an evolved robotic behaviour using a biological model of collegial decision making. In Ziemke, T., Balkenius, C. & Hallam, J. (eds.) *From Animals to Animats 12. Proceedings of the twelveth International Conference on Simulation of Adaptive Behavior, SAB*, vol. 7426 of *Lecture Notes in Computer Science*, 381–390, 10.1007/978-3-642-33093-3_38 (Springer, Berlin, Germany, 2012).

[53] Hansen N, Ostermeier A (2001). Completely derandomized self-adaptation in evolution strategies. Evolutionary Computation.

[54] Glasmachers, T., Schaul, T., Yi, S., Wierstra, D. & Schmidhuber, J. Exponential natural evolution strategies. In *Proceedings of the 12th Annual Conference on Genetic and Evolutionary Computation, GECCO*, 393–400, 10.1145/1830483.1830557 (ACM, 2010).

[55] Stanley KO, Miikkulainen R (2002). Evolving neural networks through augmenting topologies. Evolutionary Computation.

[56] Birattari, M. *Tuning Metaheuristics: A Machine Learning Perspective* (Springer, Berlin, Germany, 2009).

[57] Birattari, M. *et al*. (eds.) *Proceedings of the Genetic and Evolutionary Computation Conference, GECCO*, 11–18 (Morgan Kaufmann Publishers, San Francisco, CA, USA, 2002).

[58] Balaprakash, P. *et al*. (eds.) *Hybrid Metaheuristics, 4th International Workshop, HM 2007*, vol. 4771 of *LNCS*, 108–122, 10.1007/978-3-540-75514-2_9 (Springer, Berlin, Germany, 2007).

[59] Birattari, M., Yuan, Z., Balaprakash, P. & Stützle, T. F-Race and Iterated F-Race: an overview. In Bartz-Beielstein, T., Chiarandini, M., Paquete, L. & Preuss, M. (eds.) *Experimental Methods for the Analysis of Optimization Algorithms*, 311–336, 10.1007/978-3-642-02538-9_13 (Springer, Berlin, Germany, 2010).

[60] López-Ibáñez M, Dubois-Lacoste J, Pérez Cáceres L, Birattari M, Stützle T (2016). The irace package: iterated racing for automatic algorithm configuration. Operations Research Perspectives.

[61] Ligot A, Kuckling J, Bozhinoski D, Birattari M (2020). Automatic modular design of robot swarms using behavior trees as a control architecture. PeerJ Computer Science.

[62] Sekhavat YA (2017). Behavior tree for computer games. International Journal on Artificial Intelligence Tools.

[63] Ligot A, Birattari M (2022). Zenodo.

[64] Chambers, J. M., Cleveland, W. S., Kleiner, B. & Tukey, P. A. *Graphical Methods For Data Analysis* (CRC Press, Belmont, CA, USA, 1983).

