# Supplementary material for: On Using Simulation to Predict the Performance of Robot Swarms
Source: Sci Data. 2022 Dec 29;9:788. doi: 10.1038/s41597-022-01895-1 (PMC9800372; doi:10.1038/s41597-022-01895-1)
Supplement: Supplementary file 1 — Supplementary Figures [file 41597_2022_1895_MOESM1_ESM.pdf]

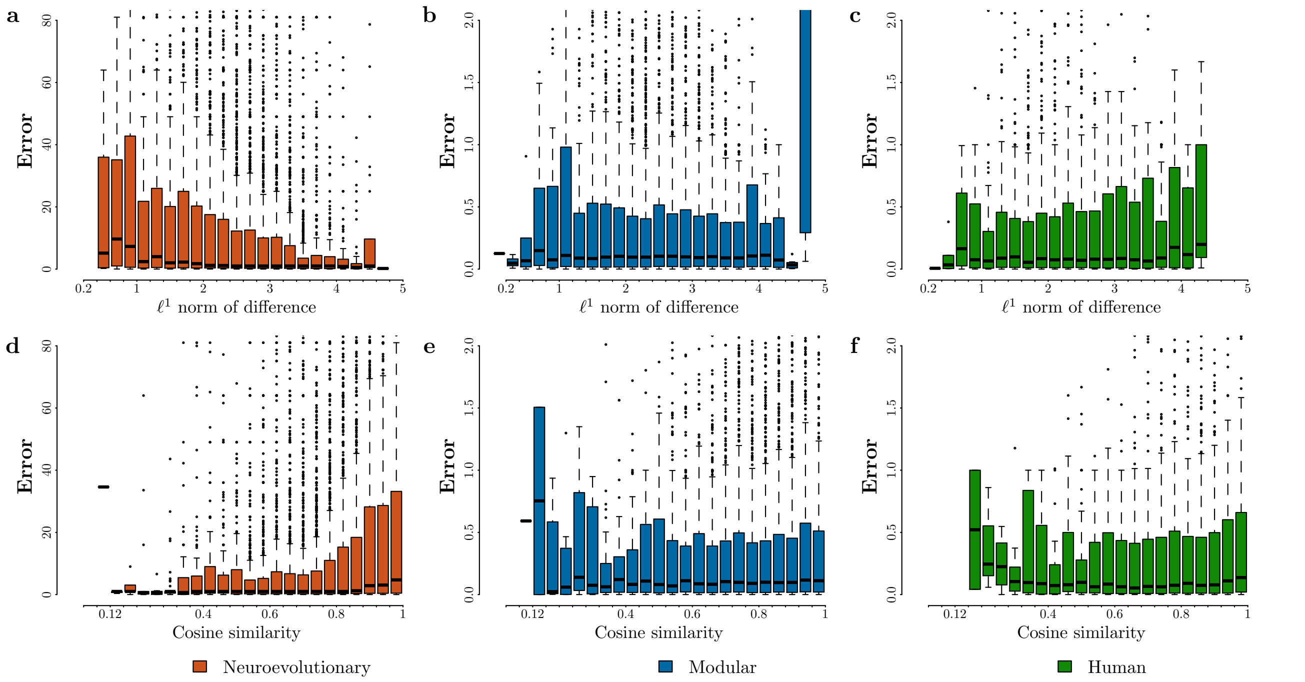

Supplementary Figure 1. Width of the pseudo-reality gap: *error* of control software produced by neuroevolutionary methods **(a, d)**, modular methods **(b, e)**, and human designers **(c, f)**. Widths are computed with the ℓ^1 norm of differences **(a, b, c)** and the cosine similarity **(d, e, f)**. Pearson correlation coefficients are equal to **(a)** -0.023 **(b)** -0.006 **(c)** -0.038 **(d)** 0.043 **(e)** 0.002 **(f)** 0.015.

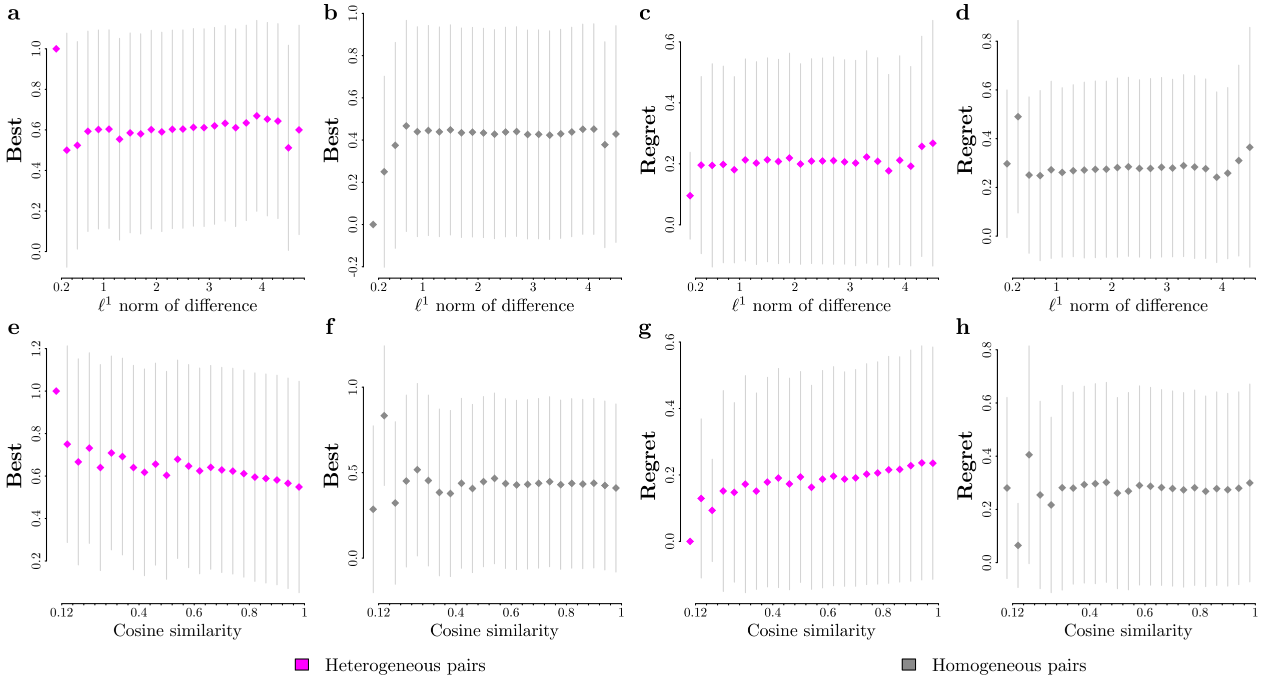

Supplementary Figure 2. Width of the pseudo-reality gap: *best* **(a, b, e, f)** and *regret* **(c, d, g, h)** when considering heterogeneous **(a, c, e, g)** and homogeneous **(b, f, d, h)** pairs of instances of control software. Widths are computed with the ℓ^1 norm of differences **(a, b, c, d)** and the cosine similarity **(e, f, g, h)**. Pearson correlation coefficients are equal to **(a)** 0.03 **(b)** -0.007 **(c)** 0.001 **(d)** 0.008 **(e)** -0.054 **(f)** -0.003 **(g)** 0.052 **(h)** -0.004.
